# Correlation between serum 25-hydroxyvitamin D levels and carotid intima-media thickness in a Brazilian population descended from African slaves

**DOI:** 10.1590/1414-431X20177185

**Published:** 2018-02-26

**Authors:** F.C. Monteiro, N.R. Mandarino, E.M. Santos, A.M. Santos, J.V. Salgado, D.J.A. Brito, B.J.L. Salgado, J.S. Lages, G. Castelo Branco, N. Salgado

**Affiliations:** 1 Serviço de Cardiologia, Hospital Universitário da Universdade Federal do Maranhão, São Luís, MA, Brasil; 2 Departamento de Enfermagem, Universidade Federal do Maranhão, São Luís, MA, Brasil; 3 Departamento de Saúde Pública, Universidade Federal do Maranhão, São Luís, MA, Brasil; 4 Departamento de Ciências Fisiológicas, Universidade Federal do Maranhão, São Luís, MA, Brasil; 5 Serviço de Nefrologia, Hospital Universitário da Universidade Federal do Maranhão, São Luís, MA, Brasil

**Keywords:** Vitamin D deficiency, Carotid intima-media thickness (C-IMT), Atherosclerosis, African continental ancestry group

## Abstract

Hypovitaminosis D has been identified as a possible new cardiovascular risk factor. However, the results of studies correlating serum vitamin D levels with markers of subclinical atherosclerosis have been conflicting. The aim of this study was to correlate serum levels of 25-hydroxyvitamin D [25(OH)D] with carotid intima-media thickness (C-IMT) and conventional cardiovascular risk factors in Afro-descendants. A cross-sectional analysis was performed on a sample of 382 individuals from a cohort of descendants of African slaves, inhabitants of “Quilombola” communities, with a mean age of 57.79 ±15.3 years, 54.5% of whom were women. Socio-demographic and clinical data were collected and biochemical tests were performed, including serum levels of 25(OH)D by electrochemiluminescence and urinary albumin excretion, evaluated by the albumin/creatinine ratio (ACR) in a spot urine sample. All participants underwent high-resolution ultrasonography for C-IMT measurement. Hypovitaminosis D was defined as serum 25(OH)D levels <30 ng/mL. The mean serum 25(OH)D levels were 50.4±13.5 ng/mL, with a low prevalence of hypovitaminosis D (4.86%). By simple linear correlation, a significant inverse association between 25(OH)D levels and C-IMT (r=-0.174, P=0.001) was observed. However, after multiple linear regression analysis, the significance of the association between serum levels of 25(OH)D and C-IMT measurement was lost (β=-0.039, P=0.318) and only male gender, age, smoking, systolic blood pressure, glucose and low density lipoprotein (LDL)-cholesterol remained significantly associated with C-IMT. Levels of 25(OH)D were independently and positively associated with HDL-cholesterol and inversely associated with age and ACR. In conclusion, no independent association between 25(OH)D levels and C-IMT was observed in this population. On the other hand, there was an inverse association with albuminuria, a marker of endothelial lesion.

## Introduction

Cardiovascular disease (CVD) is the leading cause of death and disability worldwide (1). Although the role of traditional risk factors has already been established, it is known that they may not fully explain the development of CVD. Recent evidence suggests that vitamin D deficiency, currently very prevalent, with around one billion individuals affected worldwide (2), might be associated with an increased risk of CVD (3).

Vitamin D, in fact a steroid hormone, has as its primary function the regulation of calcium and phosphorus homeostasis in interaction with the parathyroid glands, kidneys and intestines. Under normal conditions, only about 10% of the vitamin requirement is obtained by food intake, the rest of which is synthesized in the body, starting with the activation of a cutaneous precursor by solar ultraviolet radiation and involving successive hydroxylation processes at the hepatic and renal levels (4,5).

The role of vitamin D in the regulation of bone metabolism is already well established. Thus, diseases such as rickets and osteomalacia have been classically attributed to hypovitaminosis D (5). However, in recent years, several studies have demonstrated that the function of vitamin D extends well beyond bone health, being exerted on specific receptors, the vitamin D receptors (VDR), present in many cell types and modulating more than 3% of the human genome (5). Thus, hypovitaminosis D has been associated with disorders as varied as autoimmune diseases, infections and cancer. Concerning CVD, hypovitaminosis D has been independently associated with the occurrence of myocardial infarction (6), stroke (7), peripheral arterial disease (8) and cardiovascular death (3).

The mechanisms by which vitamin D levels may influence cardiovascular risk are still not fully understood and their participation in atherogenesis has been postulated. However, studies attempting to correlate hypovitaminosis D with early signs of atherosclerosis in different populations have reported conflicting findings (4,) and no research of this nature has been identified in the Latin American population.

Thus, in the present study, we sought to correlate serum levels of 25-hydroxyvitamin D [25(OH)D], the stable circulating form of the vitamin, with a marker of subclinical atherosclerosis, the measure of carotid intima-media thickness (C-IMT), and conventional risk factors in Afro-descendant individuals, inhabitants of Quilombola communities in the western coast of the State of Maranhão, Brazil.

## Material and Methods

### Study population

This cross-sectional study evaluated individuals aged ≥18 years, inhabitants of Quilombola communities in the municipality of Alcântara, west coast of the State of Maranhão, Brazil, that were participants in a prevalence study of chronic kidney disease, the PREVRENAL study (18). The 1539 individuals participating in the PREVRENAL study had been selected among the inhabitants of 32 communities through a probabilistic sampling process. All PREVRENAL participants were examined initially in the community itself, after receiving clarifications about the research objectives and signing the informed consent form. Socio-demographic, lifestyle (smoking, alcohol consumption and work activity), anthropometric (weight, height and waist circumference) and clinical (previous pathological history and blood pressure) data were collected. Blood samples, after a 12-h fast, and urine were collected for biochemical analysis and measure of albumin excretion, respectively.

All the individuals identified as having systemic arterial hypertension (SAH), diabetes mellitus (DM), albuminuria and/or reduced estimated glomerular filtration rate (GFR) were referred to a University Hospital for specialized exams and considered eligible for the present study. Thus, out of 416 participants who fulfilled this initial inclusion criterion, 25 were excluded because they presented an overt cardiovascular disease and nine did not attend to perform the complementary exams, so that, at the end, 382 individuals (91.8% of those initially eligible) constituted the sample object of the present research.

### Clinical evaluation

Standardized questionnaires were used in the initial evaluation to record information. Data on alcohol consumption (yes/no) and smoking (yes/no) were obtained. Blood pressure was defined as the mean of the last two of three measurements, performed at intervals ≥2 min, using a mercury column sphygmomanometer after sitting for at least 5 min. SAH was defined as the finding of any of the following four criteria: systolic blood pressure ≥140 mmHg, diastolic blood pressure ≥90 mmHg, previous diagnosis by a physician, or use of antihypertensive medication. The diagnosis of DM was performed according to the following criteria: use of hypoglycemic agents, fasting glycemia ≥ 126 mg/dL or an oral glucose tolerance test ≥200 mg/dL. The body mass index (BMI) was calculated using the formula: BMI=weight/height^2^ (kg/m^2^). The waist circumference was measured at the midpoint between the last rib and the iliac crest with the patient in a standing position.

### Biochemical evaluation

The biochemical measurements were automated and performed in a single laboratory. The GFR was estimated by the CKD-EPI (Chronic Kidney Disease Epidemiology Collaboration) formula, considered low if <60 mL·min^-1^/(1.73 m^2^), based on the definition of chronic kidney disease (CKD) from the National Kidney Foundation (19).

The 25(OH)D dosage was performed by means of the electrochemiluminescence fixation assay (Elecsys, Roche Diagnóstica, Brazil). A serum level ≥30 ng/mL was considered normal. Values <30 ng/mL were grouped as insufficient (20–29 ng/mL) and deficient (<20 ng/mL) (20).

Urine albumin assay was performed by immunoturbidimetry. Albuminuria was evaluated in an isolated urine sample by means of the albumin/creatinine ratio (ACR), and reported as mg/g.

### Measurement of carotid media-intima thickness (C-IMT)

The carotid artery ultrasound examination was performed with a 7.5 MHz linear transducer in longitudinal section in B mode, by a single experienced examiner, blind to the participants' clinical and laboratory information, in a GE model Vivid 3 device. The C-IMT was measured at the distal wall (farthest from the transducer) of the common carotid artery, 10 mm proximally to its bifurcation, on both sides, according to current recommendations (21). The measurement consists of the distance between two echogenic lines represented by the lumen-intima and medium-adventitia interfaces of the arterial wall, being considered normal when <0.9 mm. In the present study, the arithmetic mean of the C-IMT measurements obtained in the two carotid arteries was considered for analysis. A thickening ≥1.5 mm was categorized as an atheromatous plaque.

### Statistical analysis

The Kolmogorov Smirnov test was used to evaluate the normality of the continuous variables. Qualitative variables are reported as relative frequency (%) and continuous variables as mean and standard deviation (SD). Thechi-square test was used to compare proportions. For comparison of means, we used the Student’s *t*-test. To analyze the degree of linear correlation between continuous variables, Pearson's correlation coefficient was calculated. Associations presenting a P<0.20 at the bivariate analysis were submitted to multiple linear regression analysis. In the multiple linear regression model, gender (male=0 and female=1) and smoking (absent=0 and present=1) were included as dummy variables. The dichotomization of 25(OH)D serum levels according to the 50th percentile was chosen because of the very low prevalence of hypovitaminosis D, according to the conventional cut-off point. Data were analyzed using SPSS 18.0 for Windows (IBM, USA), adopting P<0.05 (5%) as significant.

### Ethical considerations

This research was carried out in accordance with the Brazilian National Health Council Resolution 196/96, active throughout the national territory. The inclusion of the participants was done after signing a Term of Free and Informed Consent that included the information on data confidentiality and its exclusive use in this research. The PREVRENAL project was approved by the Ethics Committee of the university institution involved in the study.

## Results

The analyzed sample consisted of 382 individuals, with a mean age of 57.79±15.39 years, being almost half with age ≥60 years, with a slight predominance of females. As expected, almost 90% of participants were non-white, and most had low income and low level of schooling. Farmers and fishermen made up half the sample. The expressive prevalence of SAH, DM and albuminuria are justified by the inclusion criteria of study participants. The mean serum levels of 25(OH)D were within the range considered healthy (≥30 ng/mL), with a low prevalence of hypovitaminosis D (4.86%). Slightly more than half of the participants had increased C-IMT and almost half had carotid atheromatous plaques. These and other characteristics of the study population are presented in Table 1.

**Table 1.** Socio-demographic, clinical, anthropometric and laboratory characteristics of the study population (n=382).

Characteristics	
Age (years)	57.79±15.39
Age ≥60 years, n (%)	181 (47.38)
Female gender, n (%)	208 (54.5)
Non-whites, n (%)	328 (86)
Occupation, n (%)	
Farmers/fishermen	194 (50.9)
Retired	133 (34.9)
Other	54 (14.2)
Income up to a minimum wage, n (%)	302 (79.06)
Up to four years of schooling, n (%)	328 (85.86)
Smokers, n (%)	41 (10.7)
Alcoholics, n (%)	109 (28.5)
Weight (kg)	62,95±12.79
Height (cm)	155±8.30
Body mass index (kg/m^2^)	26.23±5.42
Waist circumference (cm)	68.64±32.50
Systemic arterial hypertension, n (%)	241 (66.5)
Systolic blood pressure (mmHg)	148.58±28.18
Diastolic blood pressure (mmHg)	84.64±14.41
Diabetes mellitus, n (%)	59 (17.70)
Fasting glycemia (mg/dL)	119.36±54.60
Creatinine (mg/dL)	0.86±0.31
HDL-cholesterol (mg/dL)	47.87±13.57
Men	45.81±14.01
Women	49,55±13.52
Triglycerides (mg/dL)	171.77±114.21
Albumin-creatinine ratio (mg/g)	38.8±16.19
Albumin-creatinine ratio (isolated sample of urine), n (%)	
<30 (mg/g)	284 (75.53)
30–300 (mg/g)	84 (22.34)
>300 (mg/g)	8 (2.12)
25(OH)D (ng/mL)	50.4±13.5
<30 ng/mL, n (%)	18 (4.86)
<20 ng/mL, n (%)	3 (0.81)
>100 ng/mL, n (%)	3 (0.81)
Carotid intima-media thickness (mm)	0.92±0.21
Carotid intima-media thickness ≥0.9 mm, n (%)	200 (53.50)
Carotid atheromatous plaque, n (%)	168 (44.2)

Data are reported as means±SD unless otherwise indicated. 25(OH)D: 25-hydroxyvitamin D.

Table 2 presents the characteristics of the participants according to the 50th percentile of serum 25(OH)D levels. Lower levels of vitamin D were observed in women, older individuals, hypertensive individuals, diabetics, smokers, and those with higher blood pressure and higher BMI. Regarding the biochemical parameters, lower levels of 25(OH)D were associated with lower levels of creatinine, HDL-cholesterol and hemoglobin, and higher glycemia, LDL-cholesterol and albuminuria. Higher mean C-IMT was also observed among those with lower levels of 25(OH)D. There was no significant difference in the prevalence of carotid atheromatous plaques between the two subgroups of 25(OH)D levels.

**Table 2.** Characteristics of the participants according to the 50th percentile of serum 25(OH)D levels.

	<50.7 ng/L	≥50.7 ng/L	P value
Gender			
Male, n (%)	56 (33.5)	111 (66.5)	
Female, n (%)	133 (65.5)	70 (34.5)	<0.001
Age (years)	60.8±14.4	54.4±15.8	<0.001
SAH, n (%)	139 (57.7)	95 (39.4)	0.001
DM, n (%)	36 (61.0)	23 (39)	0.002
Smokers, n (%)	16 (39.0)	23 (56)	0.006
SBP (mmHg)	152.5±29.6	144.9±26.7	0.010
DBP (mmHg)	85.6±14.0	83.7±14.9	0.197
BMI (kg/m^2^)	26.7±5.4	24.4±4.2	<0.001
WC (cm)	65.7±35.4	72.2±28.4	0.054
Creatinine (mg/dL)	0.86±0.2	0.95±0.2	<0.001
Cystatin C (mg/dL)	0.35±0.18	0.38±0.18	0.671
ACR (mg/g)	47.64±11.35	20.12±2.73	0.019
Fasting glycemia (mg/dL)	125.7±60.2	114.1±49.2	0.043
HDL-cholesterol (mg/dL)	46.6±11.4	49.6±15.2	0.038
LDL-cholesterol (mg/dL)	140.0±39.2	124.5±45.3	0.001
Triglycerides (mg/dL)	159.6±97.8	141.9±88.0	0.069
Uric acid (mg/dL)	4.78±1.38	4.73±1.34	0.796
Ferritin (mg/dL)	123.6±167.0	128.4±133.5	0.760
Hemoglobin (g/dL)	13.4±1.4	14.0±1.6	<0.001
Hs-CRP (mg/dL)	0.55±1.3	0.84±4.5	0.406
C-IMT (mm)	0.96±0.2	0.88±0.2	<0.001
C-IMT ≥0.9 mm, n (%)	112 (56)	81 (40.5)	<0.001
Carotid atheromatous plaque, n (%)	88 (46.56)	76 (42.0)	0.506

Data are reported as means±SD unless otherwise indicated. 25(OH)D: 25-hydroxyivitamin D; SAH: systemic arterial hypertension; DM: diabetes mellitus; SBP: systolic blood pressure; DBP: diastolic blood pressure; BMI: body mass index; WC: waist circumference; ACR: albumin/creatinine ratio in an isolated sample of urine; HDL: high density lipoprotein; LDL: low density lipoprotein; hs-CRP: high-sensitivity C-reactive protein; C-IMT: carotid intima-media thickness. The chi-square and Student’s *t*-tests were used for statistical analyses.

After multiple linear regression analysis, lower levels of 25(OH)D remained significantly associated with the female gender. In addition, the variables creatinine and HDL-cholesterol were positively associated with 25(OH)D levels, and age, BMI and ACR were inversely associated (Table 3).

**Table 3.** Variables independently associated with serum 25(OH)D levels after multiple linear regression analysis.

Variable	β	P value
Gender	−0.246	<0.001
Age	−0.235	<0.001
BMI	−0.179	0.001
Creatinine	0.159	0.004
HDL-cholesterol	0.104	0.030
ACR	−0.235	<0.001

25(OH)D: 25-hydroxyivitamin D; BMI: body mass index; HDL: high density lipoprotein; ACR: albumin/creatinine ratio in an isolated sample of urine.

As seen in Table 4, bivariate analysis showed that an increased C-IMT was significantly associated with male gender, older age, presence of SAH, DM and smoking, as well as higher BMI, systolic blood pressure, creatinine, glycemia and LDL-cholesterol and lower levels of 25(OH)D. In a simple linear correlation analysis (Pearson), there was a significant inverse association between serum 25(OH)D levels and C-IMT (r=-0.174, P=0.001). However, after multiple regression analysis, this association lost significance when the variable age was added to the model (β=-0.039, P=0.318), so that, besides age, only male gender, smoking, and, in the positive direction, systolic blood pressure, glycemia and LDL-cholesterol levels remained significantly associated with C-IMT (Table 5).

**Table 4.** Characteristics of the participants according to carotid intima-media thickness.

	C-IMT		P value
	<0.9 mm	≥0.9 mm	
Gender, n (%)			
Male	77 (45.57)	92 (54.43)	
Female	97 (47.32)	108 (52.68)	<0.001
Age (years)	47.8±13.5	66.1±11.2	<0.001
SAH, n (%)	101 (42.44)	137 (57.56)	<0.001
DM, n (%)	19 (33.93)	37 (66.07)	0.015
Smokers, n (%)	18 (47.37)	20 (52.63)	<0.001
SBP (mmHg)	140.2±23.8	156.5±29.3	<0.001
DBP (mmHg)	84.2±13.7	85.5±14.6	0.362
BMI (kg/m^2^)	26.2±4.6	25.0±5.2	0.019
WC (cm)	70.9±31.5	66.6±33.1	0.197
Creatinine (mg/dL)	0.87±0.1	0.95±0.3	0.002
ACR (mg/g)	33.12±8.54	42.15±13.59	0.574
Fasting glycemia (mg/dL)	112.3±48.4	124.9±59.3	0.025
HDL-cholesterol (mg/dL)	47.3±13.7	48.6±13.4	0.354
LDL-cholesterol (mg/dL)	121.9±35.3	142.2±46.7	<0.001
Triglycerides (mg/dL)	151.3±108.1	154.2±87.9	0.499
Uric acid (mg/dL)	4.66±1.25	4.76±1.48	0.596
Ferritin (mg/dL)	126.4±195.2	122.8±91.8	0.825
Hemoglobin (g/dL)	13.9±1.5	13.5±1.4	0.011
hs-CRP (mg/dL)	0.81±4.5	0.57±1.2	0.499
25(OH)D (ng/mL)	52.2±14.2	48.9±12.6	0.020

Data are reported as means±SD unless otherwise indicated. C-IMT: carotid intima-media thickness; SAH: systemic arterial hypertension; DM: diabetes mellitus; SBP: systolic blood pressure; DBP: diastolic blood pressure; BMI: body mass index; WC: waist circumference; ACR: albumin/creatinine ratio in an isolated sample of urine; HDL: high density lipoprotein; LDL: low density lipoprotein; hs-CRP: high-sensitivity C-reactive protein; 25(OH)D: 25-hydroxyivitamin D. The chi-square and Student’s *t*-tests were used for statistical analyses.

**Table 5.** Variables independently associated with mean C-IMT identified by multiple linear regression analysis.

	β	P value
Age	0.618	<0.001
Gender	−0.148	0.001
Smoking	0.080	0.026
Systolic blood pressure	0.191	<0.001
Fasting glycemia	0.127	<0.001
LDL-cholesterol	0.141	<0.001

C-IMT: carotid intima-media thickness; LDL: low density lipoprotein.

## Discussion

The present study analyzed adult individuals of both genders, Afro-descendants, with cardiovascular risk factors, inhabitants of Quilombola remnant communities, and found no evidence of an independent association between serum levels of 25(OH)D and C-IMT, as well as the presence of carotid atheromatous plaques. On the other hand, an independent inverse association between 25(OH)D levels and urinary albumin excretion, assessed by ACR in an isolated urine sample, was observed.

A low prevalence of hypovitaminosis D (<5%), with only 3 cases of deficiency (0.81%), was observed in this population, with a mean serum level of 25(OH)D of 50 ng/mL. This finding differs substantially from what has been described in most studies from around the world, including tropical countries, which show that it affects in general more than half of the individuals (22). Our finding is even more striking considering that it was found in a predominantly dark-skinned population, since melanin is considered a potent natural sun blocker (22). Factors related to the lifestyle of this population, including professional activities such as farming and fishing, characterized by extensive exposure to solar radiation, probably explain this characteristic.

C-IMT is a marker of subclinical atherosclerosis widely validated as an independent predictor of cardiovascular events (23). The inverse association between serum levels of 25(OH)D and C-IMT, initially observed in this study, lost its significance when the variable age was included in the multivariate analysis model. The study corroborated, on the other hand, the role of traditional risk factors for atherosclerosis, with independent associations of the variables age, male gender, smoking, systolic blood pressure, fasting glucose and LDL cholesterol with C-IMT.

The independent inverse association between 25(OH)D levels and the urinary albumin excretion observed in this study should be highlighted. Albuminuria is considered a sensitive marker of endothelial lesion and an independent predictor of cardiovascular events (24,25). This association has also been reported in type 2 diabetics (26), in the Third National Health and Nutrition Examination Survey (NHANES III) population (27) and in individuals with CKD (28). In addition to its possible antiatherosclerotic effects (29), a nephroprotective role of vitamin D has also been suggested, and there is evidence that vitamin D therapy can reduce albuminuria (24) and delay the progression of renal disease (25).

The participation of vitamin D deficiency in the pathogenesis of atherosclerosis has been reported in numerous studies, based on the finding that low levels of 25(OH)D correlate with a higher incidence of deaths and cardiovascular events, particularly of atherosclerotic nature (3,6,7,30). This hypothesis is reinforced by the fact that a higher prevalence of obesity, dyslipidemia, metabolic syndrome, SAH and DM, which are recognized as important factors in the pathophysiology of atherosclerosis, have also been observed in individuals with hypovitaminosis D (22).

In addition, there is biological plausibility to justify the antiatherosclerotic role of hypovitaminosis D, which has been explained by several possible mechanisms (29). The active form of vitamin D, the 1,25-dihydroxyvitamin D, through its binding to VDR, regulates numerous genes involved in processes of fundamental importance in cardiovascular physiology, such as the renin-angiotensin system activity, proliferation, cell migration and differentiation, membrane transport, cell adhesion, immune response and cytokine expression. Vitamin D receptors have been found in all major cellular types of the cardiovascular system, including cardiomyocytes and especially cells involved directly in the atherosclerotic process, such as arterial wall cells (endothelial and smooth muscle cells) and immune cells. Thus, vitamin D deficiency, according to several studies, some of which experimental, may play a determining role in processes such as apoptosis, oxidative stress, smooth muscle cell proliferation, inflammation and thrombosis. Vitamin D also has a role in the mechanisms of insulin synthesis, secretion and resistance, which would justify the association between its deficiency and a higher incidence of DM (29,31,32). On the other hand, hyperparathyroidism secondary to chronic hypovitaminosis D has also been reported as an additional cardiovascular risk factor in this condition (31).

Thus, studies attempting to correlate hypovitaminosis D with subclinical atherosclerosis markers have attracted considerable interest from several researchers in recent years, with variable results. Vitamin D deficiency has been associated with endothelial dysfunction (9,10) and higher coronary calcium score (13). In the elderly, Reis et al. (4) verified an independent inverse association between serum 25(OH)D levels and internal C-IMT. In type 2 diabetics, an inverse association was also observed by Targher et al. (11) but not by Winckler et al. (12). In patients with CKD, an inverse correlation was observed by Yadav et al. (14) but not by Zang et al. (15). Finally, the two largest studies identified, one involving 926 climacteric women (16), and the other, 3.430 individuals at high cardiovascular risk (17), also found divergent results, as only the former found an association between low levels of 25(OH)D and greater C-IMT. Population differences and methodological limitations, such as the small sample size of some of them and the lack of adequate adjustment for all potential confounding factors, probably justify such discrepancies.

The positive association between 25(OH)D and HDL-cholesterol levels, which is classically linked to lower cardiovascular risk, observed in this study and also by others (17,29,33,34), deserves to be emphasized. Other favorable effects of vitamin D on the lipid profile, such as reduction of triglycerides and LDL-cholesterol, have also been described (29). The mechanisms by which vitamin D may favorably affect lipid metabolism have not yet been fully elucidated. *In vitro* studies have shown that the incubation of adipocyte culture with calcitriol promoted an increased expression and activity of lipoprotein lipase, an enzyme related to the reduction of triglyceride levels and increase in HDL-cholesterol (29).

The results of the present study, therefore, do not support the hypothesis of the antiatherosclerotic, vasculoprotective role of vitamin D, since the inverse association between levels of 25(OH)D and C-IMT did not persist in the multivariate analysis. However, our study may have had limited statistical power because of the very low prevalence of hypovitaminosis D. With a variation of 25(OH)D levels almost entirely within the normal range, it would be unlikely to find a relevant role of the vitamin in the pathogenesis of atherosclerosis in these individuals. On the other hand, its inverse association with urinary excretion of albumin should be valued and may indicate a role for lower levels of vitamin D, even though currently classified as "normal", at an earlier stage of atherosclerosis in this population. Therefore, based on our findings, the association of hypovitaminosis D with the pathogenesis of atherosclerosis in this population should not be totally discarded.

The inconsistency of results in the literature on the association between vitamin D deficiency and early markers of atherosclerosis, coupled with the divergence between observational and intervention studies, have stimulated an intense debate on the causality of hypovitaminosis D in CVD in the last decade (22,31,35). Many scholars argue that hypovitaminosis D would not play a determinant role in the pathophysiology of diseases, but would be only a marker of inflammation, hence its association with CVD. However, the remarkable limitations of previous interventional studies, such as small sample size, short period of observation, varied doses of vitamin D used, and joint administration with calcium, among others, should be emphasized. Thus, such a debate should persist until results from large, randomized ongoing trials assessing the impact of supplementation on hard outcomes, are available in the coming years.

The present study has some limitations. Its cross-sectional design does not allow establishing a causal relationship in the associations found, as well as the selection of a sample from a specific population does not allow the extrapolation of our results to other populations. In addition, the limited statistical power of the study, due to its relatively small sample size and the low proportion of individuals with hypovitaminosis D observed, should be considered. Moreover, the reproducibility of the C-IMT measurements, performed by a single experienced examiner, was not tested. On the other hand, some strengths should be highlighted. It is a study approaching a population not previously studied, that correlated serum levels of 25(OH)D not only with C-IMT, the main target of this article, but also with albuminuria, in addition to several traditional cardiovascular risk factors/markers. Finally, this seems to be the first Latin American study of this nature.

In conclusion, no independent association between serum levels of 25(OH)D and C-IMT was observed in this population. On the other hand, our data suggest an inverse association between C-IMT and albuminuria.
